# Surgical Repair of a Transannular Rupture During Transfemoral Transcatheter Aortic Valve Replacement

**DOI:** 10.1177/11795476211038126

**Published:** 2021-08-06

**Authors:** Ryaan EL-Andari, Sabin J Bozso, Jimmy JH Kang, Vinod K Manikala, Michael C Moon, Mohammed Al-Aklabi, Robert C Welsh, Jeevan Nagendran

**Affiliations:** 1 Faculty of Medicine and Dentistry, University of Alberta, Edmonton, AB, Canada; 2 Division of Cardiac Surgery, Department of Surgery, University of Alberta, Edmonton, AB, Canada; 3 Division of Cardiology, Department of Medicine, University of Alberta, Edmonton, AB, Canada

**Keywords:** Transcatheter aortic valve replacement, annular rupture, aortic valve replacement

## Abstract

Annular rupture is a rare but life-threatening complication of transcatheter aortic valve replacement (TAVR). Mortality rates are high if immediate intervention, most often necessitating surgical repair, is not performed. Herein, we describe an 87-year-old man who, after deployment of TAVR, experienced acute decompensation and required urgent conversion to a midline sternotomy to repair an aortic annular rupture. This case demonstrates an example of a rare but severe complication of TAVR. This report provides an in-depth description of the surgical approach to repair an aortic annular rupture and demonstrates the utility of performing minimally invasive procedures inside a hybrid operating room.

## Introduction

Since the advent of transcatheter aortic valve replacement (TAVR) in 2002, the indications for TAVR have been expanding and its use increasing.^1^ TAVR has allowed for improvement in quality of life and survival in previously inoperable aortic stenosis (AS) patients as well as those at high, intermediate, and low risk for traditional surgical intervention.^1^ One of the most dangerous and rare complications is aortic annular rupture occurring in <1% of all TAVR procedures. Proposed risk factors include valve oversizing, calcification of the left ventricular (LV) outflow tract (LVOT), and post dilation of the deployed TAVR.^2-7^ Uncontained annular rupture progresses to tamponade, hemodynamic collapse, and death.^2,5^ Repair of annular rupture varies from conservative approaches such as pericardial drainage to sternotomy with aortic valve replacement and surgical repair of the rupture site.^3,5,6^ Herein, we describe a patient who required urgent conversion to a midline sternotomy in order to repair an annular rupture during TAVR.

## Case History

An 87-year-old man presented with NYHA class III symptoms was found to have severe AS and was subsequently referred for TAVR.

Past medical history included repair of inguinal hernias in 2011, left knee arthroplasty in 2013, and prior diagnosis of atrial fibrillation and benign prostatic hypertrophy. The patient’s preoperative STS risk score for mortality was 4.3% and Euroscore II was 1.93%.

### Investigations

**Figure 1.**
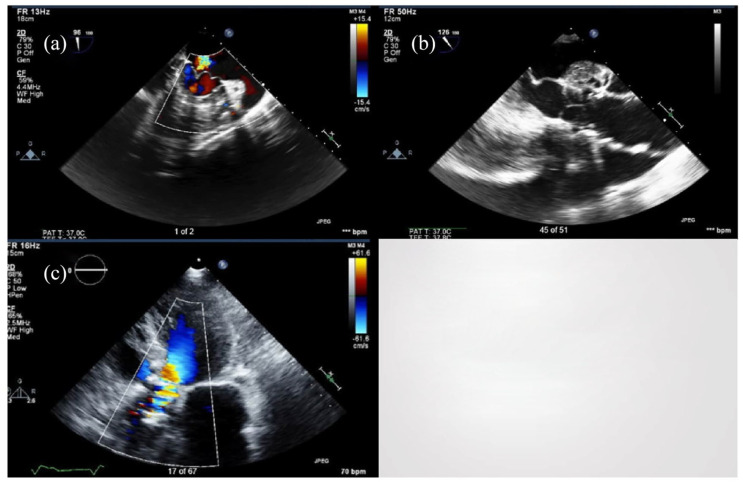
Preoperative echocardiogram of the aortic valve (a and b) and a postoperative echocardiogram of the aortic valve (c).

Coronary angiography demonstrated 50% stenoses in the mid-left anterior descending and proximal left circumflex arteries. Preoperative echocardiography demonstrated mild-moderate mitral annular calcification, tricuspid valve calcification, mild-moderate tricuspid regurgitation, and severe LV and left atrial dilation. Estimated LV ejection fraction (LVEF) was >55%. Preoperative echocardiographic images are displayed in Figure 1. Imaging of the AV identified severe thickening and restricted leaflet range of motion, severe AS, mild aortic regurgitation (AR), AV maximum gradient of 85.7 mmHg, mean gradient of 51.1 mmHg, and area of 0.74 cm^2^. Computed Tomography scan demonstrated an aortic annulus size 29 × 21 mm and 5.4 cm^2^ with leaflet calcification, mixed atherosclerotic disease within the ascending aorta and proximal great vessels without flow-limiting stenosis. No LVOT or aortic annular calcification was identified.

### Treatment and outcomes

The patient was brought to the hybrid operating room for planned TAVR. A 14 French sheath was placed in the right femoral artery with left femoral artery and vein supporting access. A 26 mm Sapien S3 (Edwards Lifesciences, Irvine, CA) TAVR was deployed at nominal pressure. Immediately following deployment, the patient became hypotensive and hemodynamically unstable. Cardiopulmonary resuscitation (CPR) was initiated with rapid endotracheal intubation and ventilation. Intraoperative transthoracic echocardiogram (TTE) identified rupture of the posterior aortic root with clot formation, large pericardial effusion, and cardiac tamponade. Aortic root angiogram demonstrated superior strut of the valve was extravascular. A pericardial drain was placed percutaneously which drained bright red blood. Based on TTE, fluoroscopy, and clinical scenario, a presumed diagnosis of annular rupture was made.

After Heart Team review, it was deemed reasonable to proceed with open exploration and repair of presumed annular rupture. The 14 French Edwards E Sheath (Edwards Lifesciences, Irvine, CA) in the right common femoral artery was replaced with a 17 French arterial cannula and connected to the cardiopulmonary bypass (CPB) circuit after systemic heparinization. The 7 French sheath in the left common femoral vein was replaced with a 25 French Medtronic multiport venous cannula (Medtronic, Dublin IE) placed in the right atrium. This was performed during CPR, active removal of blood through the percutaneous pericardial drain, and autotransfusion.

Once the activated clotting time was >400, CPB was initiated with active cooling. A midline sternotomy was performed during cooling and the pericardium opened once the heart fibrillated at 26°C. A superior vena cava cannula was placed and connected to the venous aspect of the CPB circuit via Y shape connector. An LV vent was placed in the right superior pulmonary vein and retrograde cardioplegia cannula placed in the coronary sinus. Once the patient’s blood temperature reached 18°C deep hypothermic circulatory arrest was established. The aorta was opened and resected to the level of the innominate artery due to dissection of the ascending aorta. Simultaneously, retrograde cardioplegia was administered via the coronary sinus. A 30 mm graft with an 8 mm side limb was anastomosed to the aortic hemi-arch with a running 4-0 Prolene suture. An arterial CPB cannula was placed in the 8 mm side limb. The graft was clamped and antegrade flow restored. The total circulatory arrest time was 11 minutes. The aorta was resected to the level of the sinotubular junction. The TAVR valve was removed and native valve excised. A ventricular septal perforation (VSP) below the right coronary cusp and a tear below the noncoronary cusp were identified. The VSP and free wall rupture were repaired primarily with pledgeted 4-0 and 3-0 Prolene sutures. A 21 mm Perimount Magna Ease valve (Edwards Lifesciences, Irvine, CA) was sized to the annulus and secured with 12 pledgeted Ethibond sutures. The 30 mm graft was then anastomosed to the sinotubular junction. A warm dose of antegrade cardioplegia was given and the aortic cross-clamp removed. The heart regained sinus rhythm spontaneously and pacing wires were placed.

The anastomosis of the graft to the sinotubular junction appeared to be tearing. Therefore, the graft was re-cross clamped, the heart rearrested, and the graft opened. The proximal anastomosis was repaired. A warm dose of cardioplegia was given and the cross-clamp removed. The patient was weaned from CPB and protamine administered. Bright red blood emerged between the aorta and SVC at the aortic annulus underneath the noncoronary sinus at the level of the aorto-mitral curtain. The decision was made to re-heparinize, re-cross-clamp, and rearrest the heart. The implanted valve was removed. The tear in the annulus underneath the noncoronary sinus, which we previously attempted to repair with pledgeted Prolene sutures, had torn open widely. We, therefore, elected to reconstruct the annulus with a bovine pericardial patch. A running 4-0 Prolene suture was then used to sew the bovine pericardial patch to the aortic annulus and aorta. The newly reconstructed aortic annulus was then sized for a 19 mm Perimount Magna Ease valve (Edwards Lifesciences, Irvine, CA). Interrupted pledgeted Ethibond sutures were used to secure the valve at the neo-aortic annulus. The 30 mm graft was then anastomosed to this reconstructed sinotubular junction and the proximal 30 mm graft to the distal 30 mm graft. A warm dose of cardioplegia was given and the cross-clamp was removed. The patient was weaned from CPB without difficulty and protamine was administered. Hemostasis was achieved after 3 hours of packing. The sternum was then closed with stainless steel wires. Subcutaneous tissue and skin were closed in standard fashion. The patient was brought back to the cardiac surgery intensive care unit (ICU) in critical but stable condition. Postoperatively, the patient experienced an acute kidney injury, peripheral edema, and a sore throat post-intubation. The patient required inotropes and vasopressors for 6 days postoperatively after which the patient was transferred to the cardiac ward for 7 days of additional recovery. An echocardiogram 9 days postoperatively demonstrated, an LVEF of 55-60%, trace AR, and a mean AV gradient of 29 mmHg. Postoperative images are displayed in Figure 1. After a total 13 day course in the hospital, the patient was discharged. He is currently alive and well at home, several weeks after discharge.

## Discussion

Following annular rupture, there is often rapid decompensation over minutes and a high mortality rate if untreated. Therefore, it is imperative that annular rupture be prevented and addressed appropriately when it occurs. Preoperative assessment via CT scan to identify aortic annular size aids in preventing valve oversizing. Additionally, annular and LVOT calcification should be identified as they increase the risk of annular rupture. Intraoperatively, care should be taken in placement of the TAVR. Postdilation increases the risk of rupture and should be handled with care if performed. In this case, the patient did not present with annular calcification, the implanted valve was not oversized, and postdilation was not performed demonstrating the occurrence of annular rupture even when not anticipated.

Treatment varies from supportive measures with pericardial drainage to sternotomy in order to replace the TAVR valve and repair the rupture site. As patients rapidly decompensate, treatment of annular rupture must be performed immediately. The utilization of a heart team and a hybrid operating room allows for surgical intervention when required, as illustrated by this case, with successful sequential procedural staging and clinical decision making inside of a hybrid operating room. Following urgent resuscitation and rapid pericardiocentesis improved hemodynamic stability allowed controlled initiation of CPB and patient cooling with circulatory arrest prior to opening the pericardium. Following extensive open surgical repair, the patient recovered without sustained complication with a total length of stay of less than 2 weeks.

## Conclusions

The increased prevalence of TAVR has allowed for previously inoperable patients and those at high, intermediate, and low surgical risk to undergo valve replacement improving survival and quality of life. This case demonstrates a dangerous, albeit rare, complication of TAVR describing the acute decompensation of a patient experiencing annular rupture, and the successful surgical repair undertaken to correct the annular rupture.
